# Correction: Investigation of a flexible, room-temperature fiber-shaped NH_3_ sensor based on PANI–Au–SnO_2_

**DOI:** 10.1039/d5ra90009c

**Published:** 2025-01-24

**Authors:** Qiuning Wang, Yuan Peng, Bin Guo, Jianhai Sun, Yaxia Liu, Yanjun Wang, Hongyan Zhang

**Affiliations:** a Beijing Key Laboratory of Clothing Materials R&D and Assessment, Beijing Engineering Research Center of Textile Nanofiber, Beijing Institute of Fashion Technology Beijing 100029 P. R. China zhyzzh@126.com; b Zhongshan Institute of Changchun University of Science and Technology P. R. China; c State Key Laboratory of Transducer Technology, Aerospace Information Research Institute, Chinese Academy of Sciences Beijing 100194 P. R. China; d School of Fashion, Beijing Institute of Fashion China; e Beijing Institute of Fashion Technology, School of Fashion Flat Knitting Machine Lab China

## Abstract

Correction for ‘Investigation of a flexible, room-temperature fiber-shaped NH_3_ sensor based on PANI–Au–SnO_2_’ by Qiuning Wang *et al.*, *RSC Adv.*, 2024, **14**, 38530–38538, https://doi.org/10.1039/D4RA06915C.

The authors regret that there was an error in the sentence on lines 10–11 of the left column on page 38535, in the second paragraph of section 3.4. The text originally read: ‘The responses to CO, H_2_S, NO_2_, NH_3_, SO_2_, and H_2_ equaled 1.04, 1.05, 1.00, 1.12, 0.10, and 0.20, respectively.’ This sentence should read: ‘The responses to H_2_, NH_3_, CO, and SO_2_ equaled 0.20, 1.12, 0, and 0.10, respectively.’

A higher resolution version of Fig. 7 has also been included.

**Fig. 1:**
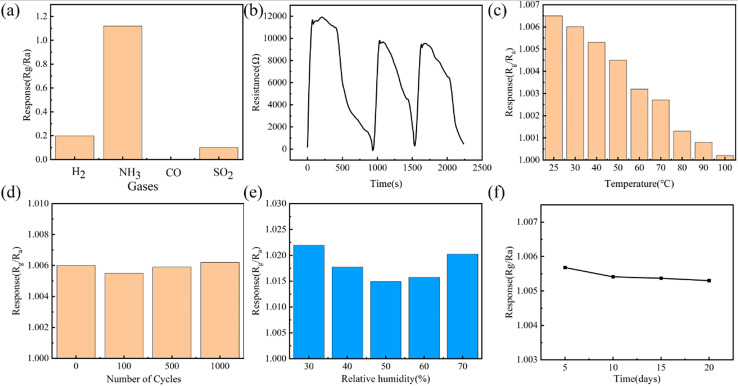
(a) Response of fiber-shaped sensor towards different interference gases with a concentration of 40 ppm. (b) Cycling response toward 40 ppm NH_3_ at room temperature. (c) Response value of the NH_3_ sensor at different operating temperatures. (d) Response changes of NH_3_ sensors after different bending cycles. (e) Response values of the NH_3_ sensor at different relative humidities. (f) Temporal stability of the NH_3_ sensors.

The Royal Society of Chemistry apologises for these errors and any consequent inconvenience to authors and readers.

